# Unravelling consensus genomic regions conferring leaf rust resistance in wheat via meta‐QTL analysis

**DOI:** 10.1002/tpg2.20185

**Published:** 2021-12-17

**Authors:** Aduragbemi Amo, Jose Miguel Soriano

**Affiliations:** ^1^ State Key Laboratory of Crop Stress Biology for Arid Areas College of Agronomy Northwest A&F Univ. Yangling Shaanxi China; ^2^ Sustainable Field Crops Programme Institute for Food and Agricultural Research and Technology (IRTA) Lleida 25198 Spain

## Abstract

Leaf rust, caused by the fungus *Puccinia triticina* Erikss (Pt), is a destructive disease affecting wheat (*Triticum aestivum* L.) and a threat to food security. Developing resistant cultivars represents a useful method of disease control, and thus, understanding the genetic basis for leaf rust resistance is required. To this end, a comprehensive bibliographic search for leaf rust resistance quantitative trait loci (QTL) was performed, and 393 QTL were collected from 50 QTL mapping studies. Afterward, a consensus map with a total length of 4,567 cM consisting of different types of markers (simple sequence repeat [SSR], diversity arrays technology [DArT], chip‐based single‐nucleotide polymorphism [SNP] markers, and SNP markers from genotyping‐by‐sequencing) was used for QTL projection, and meta‐QTL (MQTL) analysis was performed on 320 QTL. A total of 75 MQTL were discovered and refined to 15 high‐confidence MQTL (hcmQTL). The candidate genes discovered within the hcmQTL interval were then checked for differential expression using data from three transcriptome studies, resulting in 92 differentially expressed genes (DEGs). The expression of these genes in various leaf tissues during wheat development was explored. This study provides insight into leaf rust resistance in wheat and thereby provides an avenue for developing resistant cultivars by incorporating the most important hcmQTL.

## Core Ideas

- Meta‐QTL (MQTL) analysis is an effective approach to synthesize QTL information.
- MQTL analysis reduced the confidence intervals for leaf rust resistance.
- MQTL allowed the identification of candidate genes.

**Abbreviations**
AIC: Akaike information criterion
AUDPC: area under the disease progress curve
CI: confidence interval
DArT: diversity arrays technology
DEG: differentially expressed gene
DS: disease severity
EA+: early aborted colonies, without plant cell necrosis
EA−: early aborted colonies, associated with plant cell necrosis
EST+: established colonies, without plant cell necrosis
EST−: established colonies, associated with plant cell necrosis
GO: gene ontology
hcmQTL: high‐confidence MQTL
HR: host reaction
IE: infection efficiency
IR: infection rate
IT: infection type
LIA: leaf infected area
LP: latent period
LR: leaf rust resistance
LS: lesion size
LTN: leaf tip necrosis
MAS: marker‐assisted selection
MDS: maximum disease severity
MQTL: meta‐quantitative trait loci
NEC: all necrotic colonies
PVE: phenotypic variation explained
RLK: receptor‐like kinase
SNP: single‐nucleotide polymorphism
SPL: spore production per lesion
SPS: spore production per unit of sporulating tissue
SSR: simple sequence repeat.

## 1 INTRODUCTION

Leaf rust, caused by the fungal pathogen *Puccinia triticina* Erikss (Pt), causes a significant reduction in grain yield worldwide, and thus, it is considered a disease with significant importance in wheat (*Triticum aestivum* L.) (Khan et al., 2013; Kolmer, 2005). Compared with other fungal rust diseases, such as stem and stripe rust, leaf rust occurs more frequently and has a wider distribution (Bolton et al., 2008). This wide spread of leaf rust may be because the spores of *P*. *triticina* are transported long distances via wind or humans, thereby causing damage to wheat crops outside their environment or country of origin, as has been observed in various studies in North America (Bolton et al., 2008; Kolmer, 2005; Brown & Hovmøller, 2002). The most economical, efficient, environmentally sustainable, and socially acceptable way to manage rust disease globally is to grow rust‐resistant cultivars (McIntosh et al., 1995; Wiesner‐Hanks & Nelson, 2016). Therefore, to understand how best to combine genes and effectively carry out marker‐assisted selection (MAS), mapping the target genes conferring resistance in existing parental stocks is essential (Kuchel et al., 2007).

Quantitative trait loci (QTL) mapping is an effective analytical method for studying and manipulating complex traits in crops (Doerge, 2002; Xin et al., 2020). However, QTL mapping has several limitations based on the type of markers, the influence of the environment, the use of different parents, and the size of mapping populations. Therefore, little progress has been made regarding fine mapping and QTL cloning in wheat because of the paucity of high‐resolution linkage maps, as the use of simple sequence repeat (SSR) markers is constrained by their inability to saturate the wheat genome (Somers et al., 2004). Consequently, QTL are frequently present in large genomic regions, and MAS is restricted to small numbers of validated markers (Wang et al., 2015). In recent years, a revolution in the QTL analysis of complex traits has occurred via the introduction of different high‐throughput sequencing and genotyping technologies, thus aiding genetic map construction and marker development (Wang et al., 2018). Numerous QTL studies have been performed in wheat; however, the detected QTL often do not overlap either in part or in whole because of a different combination of parental lines or studies in multiple environments (Rong et al., 2007). Therefore, there is a need to detect the most promising consensus QTL found among studies using different parents that is stable across environments.

Furthermore, recognizing robust and reliable QTL and refining their intervals can be achieved by meta‐QTL (MQTL) analysis without using expensive resources (Goffinet & Gerber, 2000). The free software BioMercator (Arcade et al., 2004), used for MQTL analysis, allows the compilation of a vast number of genetic maps from various sources and can project QTL to a consensus or reference map (Veyrieras et al., 2007). Thus, MQTL analysis could identify the consensus QTL associated with the trait in multiple environments and genetic backgrounds (Goffinet & Gerber, 2000). Quantitative trait loci for similar traits may be combined synergistically into MQTL traits by a single MQTL analysis. The reported MQTL method can be used for MAS (Maccaferri et al., 2015; Yu et al., 2014).

Several studies on MQTL analysis for disease resistance in wheat have been carried out successfully, including those for tan spot resistance (Liu et al., 2020), Fusarium head blight resistance (Liu et al., 2009; Löffler et al., 2009; Venske et al., 2019; Zheng et al., 2020), leaf rust resistance (Soriano & Royo, 2015), and stem rust resistance (Yu et al., 2014). The first MQTL analysis on leaf rust focused only on identifying consensus genomic regions for the trait. In addition, the authors did not use single‐nucleotide polymorphism (SNP)‐based genetic maps because of the lack of QTL studies in leaf rust using high‐throughput SNP arrays several years ago. However, in this study, we aimed to delve deeper into the genetic architecture underlying leaf rust disease by discovering putative candidate genes from the newly released wheat genome sequence (The International Wheat Genome Sequencing Consortium et al., 2018), incorporating transcriptomic studies, and studying the function of these in genes in different tissues.

**Core Ideas**

- Meta‐QTL (MQTL) analysis is an effective approach to synthesize QTL information.
- MQTL analysis reduced the confidence intervals for leaf rust resistance.
- MQTL allowed the identification of candidate genes.

## 2 MATERIALS AND METHODS

### 2.1 Bibliographic search for wheat leaf rust resistant QTL

Using Google Scholar (https://scholar.google.com/) and the Web of Science (http://www.webofknowledge.com/), an exhaustive search for publications containing QTL conferring leaf rust resistance in wheat was performed. For each study, the information collected during the QTL compilation included the following: (a) the mapping population type of F_2:3_, recombinant inbred lines and double haploids; (b) 19 disease resistance traits including disease severity (DS), area under the disease progress curve (AUDPC), leaf infected area (LIA), infection type (IT), infection rate (IR), leaf rust resistance (LR), leaf tip necrosis (LTN), latent period (LP), spore production per unit of sporulating tissue (SPS), spore production per lesion (SPL), infection efficiency (IE), lesion size (LS), early aborted colonies, associated with plant cell necrosis (EA−), early aborted colonies, without plant cell necrosis (EA+), established colonies, associated with plant cell necrosis (EST−), established colonies, without plant cell necrosis (EST+), all necrotic colonies (NEC), maximum disease severity (MDS), and host reaction (HR); (c) the number of lines in the mapping population; (d) the logarithm of the odds score; (e) the *R* ^2^ value, which denotes the percentage of the phenotypic variation explained (PVE); and (f) the markers flanking the QTL position (Supplemental Table S1).

### 2.2 QTL projection on the consensus map

To project the largest number of QTL, the high‐density consensus map developed by Venske et al. (2019) was used. This consensus map incorporated three marker types: SNP, diversity array technology [DArT], and SSR markers. The SNPs were sourced from chip‐based markers and genotype‐by‐sequencing (Cavanagh et al., 2013; Saintenac et al., 2013; Wang et al., 2014). The SSR markers, including functional markers, were provided from three genetic maps (Wheat, Consensus SSR 2004, Wheat Composite 2004, and Wheat Synthetic × Opata) obtained from the GrainGenes database (https://wheat.pw.usda.gov/GG3/). Wheat consensus map version 4.0, which contains more than 100,000 DArT sequencing markers and nearly 4,000 DArT markers developed from over 100 genetic maps, was downloaded from https://www.diversityarrays.com/technology-and-resources/genetic-maps. The initial QTL were projected following the approach described in Chardon et al. (2004) using BioMercator v4.2 software (Arcade et al., 2004) (https://urgi.versailles.inra.fr/Tools/BioMercator-V4). Before projecting onto the consensus map, a confidence interval (CI) of 95% was homogenized across the different studies using the following formulas: 530/(*N* × PVE) for F_2:3_, 163/(*N* × PVE) for recombinant inbred lines, and 287/(*N* × PVE) for double haploids (Darvasi & Soller, 1997; Guo et al., 2006), where *N* is the number of genotypes in the mapping population, and PVE is the phenotypic variance explained by the QTL.

### 2.3 MQTL analysis and validation using genome‐wide association studies

The MQTL analysis was conducted using the software BioMercator (Arcade et al., 2004; Sosnowski et al., 2012) by incorporating two approaches for the analysis. The first approach, proposed by Goffinet and Gerber (2000), is used when the QTL count for a chromosome is <10. The second approach, proposed by Veyrieras et al. (2007), is used when the QTL count for a chromosome is above >10. For the first approach, the lowest Akaike information criterion (AIC) value was selected as the best fit model. However, the best model was selected from the AIC, AICc, AIC3, Bayesian information criterion, and average evidence weight models from the second approach. Therefore, the model with the lowest criteria in at least three of the models was selected and regarded as the best fit model. The sequences of the flanking markers of each MQTL were submitted to BLAST analysis against the Chinese Spring reference genome (https://wheat.pw.usda.gov/blast) (The International Wheat Genome Sequencing Consortium et al., 2018), and their corresponding physical positions were identified. In addition, the MQTL found in this study were validated using recent association studies with different genetic backgrounds and environments aimed at identifying loci and QTL related to leaf rust resistance in wheat.

### 2.4 Establishment of hcmQTL and candidate gene mining

To further refine the MQTL, those with at least five overlapping QTL having a physical distance <20 Mb and a genetic distance <10 cM were selected and called high‐confidence MQTL (hcmQTL). The annotated reliable genes (HighConfidenceGenes v1.1) within the interval of each hcmQTL were obtained, and their functional annotations were examined (https://wheat‐urgi.versailles.inra.fr/Seq‐Repository/Annotations).

### 2.5 Expression of candidate genes within hcmQTL intervals

To check for the candidate genes that were differentially expressed within the hcmQTL intervals, three expression datasets, NCBI‐ID ERP013983, SRP041017, and ERP009837 (Dobón et al., 2016; Rudd et al., 2015; Zhang et al., 2014) were used based on experiments reported at ExpVIP (http://www.wheat‐expression.com) (Borrill et al. et al., 2016). The ERP013983 dataset consists of differential expression data of wheat resistance cultivar Avocet inoculated with a PST 87/66 strain, with leaf samples collected at 0, 1, 2, 3, and 5 d postinoculation. In the second dataset, SRP041017, the transcriptome of the hexaploid wheat line N9134 inoculated with the Chinese *Pst* race CYR 31 was compared with the same line inoculated with powdery mildew (*Blumeria graminis* f. sp. *tritici*) race E09 at 1, 2, and 3 d postinoculation. The third dataset, ERP009837, consists of differential expression data of the wheat cultivar Riband inoculated with the fungus *Zymoseptoria tritici* (*Septoria tritici* blotch), and the expression data were collected at 1, 4, 9, 14, and 21 d postinoculation. The count data of all the expression data were further analyzed, the log_2_(fold change) was obtained using the R package Deseq2, and its correction was performed using the R package Apeglm (Zhu et al., 2019). For the DEGs discovered within the refined hcmQTL, gene ontology (GO) analysis was performed using the GENEDENOVO cloud platform (https://www.omicshare.com/tools/).

Subsequently, to identify the expression of the reported DEGs in the wheat tissues, the transcriptomics data of cultivar Azhurnaya 209‐sample RNA sequencing project, which examined the developmental timeline of commercial cultivars using a comprehensive array of samples from 24 tissue types (Ramírez‐González et al., 2018), were used in this study. The stages and their corresponding tissues are as follows: seedling stage, which includes radicle, coleoptile, stem axis, first leaf sheath, first leaf blade, first leaf blade, root, and shoot apical meristem; three leaf stage, which includes third leaf blade and third leaf sheath; tillering stage, which includes first leaf sheath, first leaf blade, shoot axis, and shoot apical meristem; full boot, which includes flag leaf sheath and flag leaf blade; ear emergence, which includes flag leaf sheath and flag leaf blade; anthesis, which includes flag leaf blade night (−0.25 h) 06:45 and fifth leaf blade night (−0.25 h) 21:45; milk grain stage, which includes flag leaf sheath, flag leaf blade, and fifth leaf blade (senescence); dough, which includes flag leaf blade (senescence); and ripening, which includes flag leaf blade (senescence). Transcripts per million (TPM) values were used to assess the candidate genes' level of expression within the hcmQTL displayed on the heat map using log_2_(TPM + 1).

## 3 RESULTS

### 3.1 QTL compilation and projection on the consensus map

A comprehensive search for QTL conferring resistance to leaf rust resulted in 50 articles published from 1999 to 2020 (Supplemental Table S1). A total of 393 QTL widely distributed across the genome were collected (Supplemental Table S2).

**FIGURE 1:**
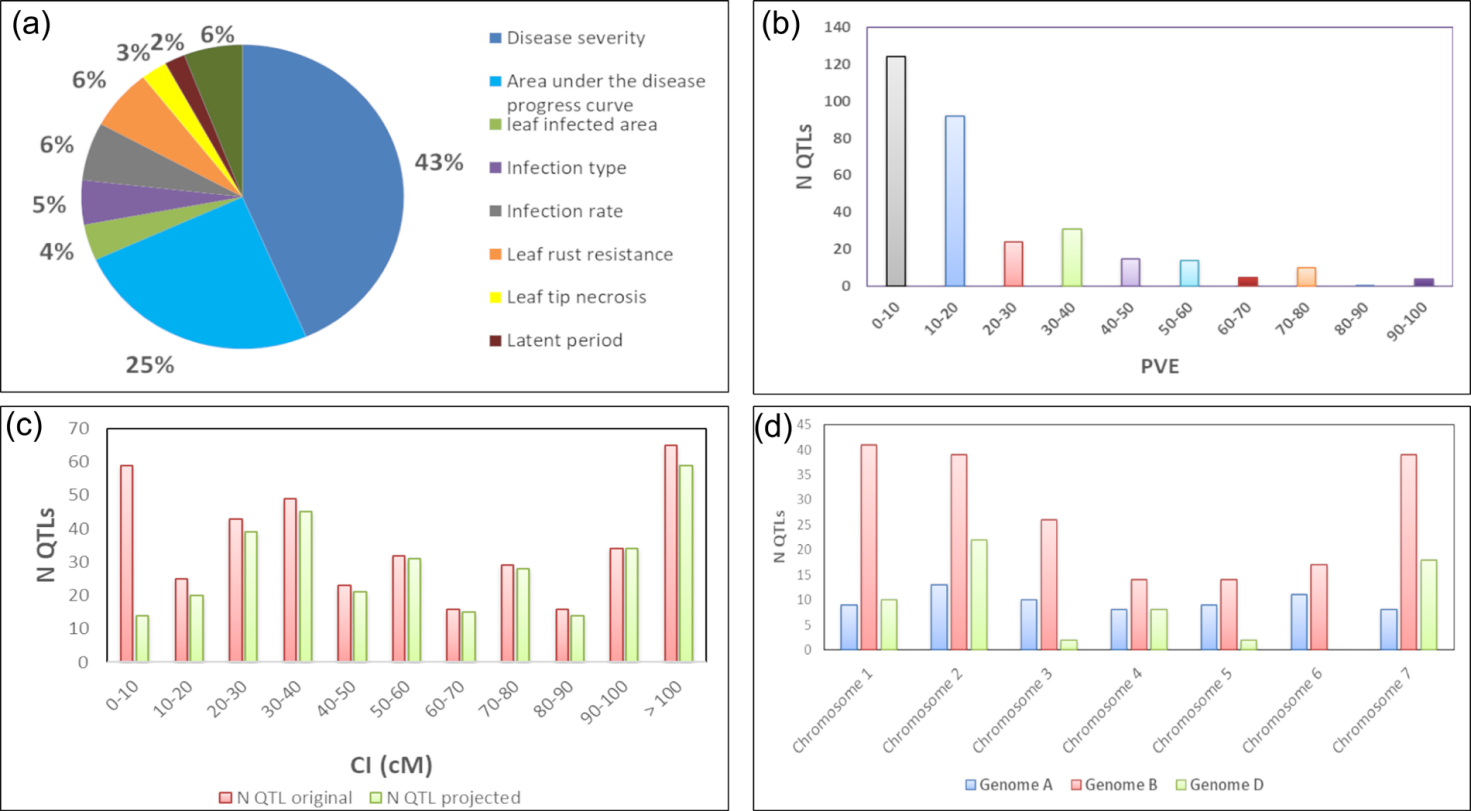
Summary information of quantitative trait loci (QTL) projected for meta‐QTL (MQTL) analysis. Frequency distribution of the number of QTL by (a) type of resistance trait conferred by the QTL; (b) phenotypic variation explained (PVE/*R* ^2^); (c) confidence interval; and (d) number of QTL per chromosome

Of the 393 QTL found, only 320 QTL had flanking markers and were thus used for MQTL analysis, leaving 73 QTL with no flanking markers on the consensus map (Supplemental Table S2). The QTL were then projected onto the consensus map constructed by Venske et al. (2019). The highest number of QTL (264) was projected on the B genome, while the D genome harbored the lowest number of QTL (59) (Figure 1). For the A genome, chromosome 2A had the highest number of projected QTL, while chromosomes 4A and 7A equally had the lowest (8). Chromosome 1B had the highest number of projected QTL (41) for the B genome, while chromosomes 4B and 5B both had the lowest (14). The overall number of QTL projected on the D genome was relatively low compared with other genomes, with the highest number of QTL (22) projected on chromosome 2D, while 6D did not harbor any projected QTL. When the trait was considered, a large proportion of the projected QTL were for disease severity (43%), followed by AUDPC (24%) (Figure 1). The rest of the trait categories tagged ‘others,’ comprised 11 traits (SPS, SPL, IE, LS, EA−, EA+, EST−, EST+, NEC, MDS, and HR). The PVE varied from 0.01 to 0.97, with 69% of the QTL reporting a PVE value <0.20. Confidence intervals ranged from 1.14 to 173.11 cM, with an average of 63.98 cM. Most of the QTL reported a CI <20 cM (81%), with 57% of the QTL showing a CI <10 cM. Only 2% of the QTL showed a CI >50 cM.

### 3.2 Unravelling consensus regions via MQTL analysis

For the MQTL analysis, the Veyrieras approach (Veyrieras et al., 2007) was used to analyze all the linkage groups except for chromosomes 1A, 4A, 5A, 7A, 3D, 4D, and 5D because they had <10 QTL; thus, the approach of Goffinet and Gerber (2000) was used for their analysis. Overall, 75 MQTL were discovered and distributed across all the chromosomes (Figure 1; Table 1). For the reported MQTL, the CI ranged from 0.03 to 25.23 cM with an average of 5.36 cM. For the A genome, 24 MQTL were found, with the highest number of MQTL (4) on chromosomes 1A, 5A, and 7A, while chromosomes 2A, 3A, 4A, and 6A harbored three MQTL. For the B genome, a total of 33 MQTL were found, representing the genome with the highest number of MQTL. Chromosome 2B reported the highest number of MQTL (8), followed by six MQTL on chromosomes 1B and 6B, and chromosomes 3B and 4B had the lowest number, with three MQTL. For the D genome, a total of 18 MQTL were found, with chromosomes 1D, 2D, and 7D harboring the most MQTL (4), while chromosomes 3D, 4D, and 5D harbored the fewest MQTL (2). The physical position of all the MQTL was computed. The mean physical confidence interval of the MQTL was 27.47 Mb, which ranged from 0.55 (MQTL1D.1) to 765.3 Mb (MQTL2B.7). The MQTL7B.4 incorporated the highest number of original QTL. From the MQTL discovered, the physical interval of six MQTL was shown to overlap, namely, MQTL1B.2 (636–648 Mb) and MQTL1B.3 (646–662 Mb), MQTL7B.3 (734–744 Mb) and MQTL7B.4 (724–750 Mb), and MQTL5B.4 (634–712 Mb) and MQTL5B.3 (685–704 Mb). Interestingly, none of the MQTL whose physical intervals overlapped did it in the genetic map.

**TABLE 1:** The meta‐quantitative trait loci (MQTL) for leaf rust resistance detected in this study

**MQTL**	**Chr** **^a^**	**Peak**	**CI** **^b^**	**Flanking markers**	**CI**	**No. QTL**	**Traits** **^c^**
			cM		Mb		
MQTL1A.1	1A	3.0	0.0–8.8	2325584–BS00026003_51	2.9–6.0	2	DS, AUDPC
MQTL1A.2	1A	25.2	24.2–26.3	wsnp_Ex_c2868_5293115–1150566	7.2–10.5	2	AUDPC
MQTL1A.3	1A	57.7	55.7–59.8	4909851–1151824	365.6–449.6	2	DS, IR
MQTL1A.4	1A	125.6	122.6–128.6	wPt‐1792–Xbarc158	569.9–593.3	1	AUDPC
MQTL1B.1	1B	36.4	35.4–37.3	1770278–1108935	29.6–48.1	6	LR, LIA, IT, AUDPC
MQTL1B.2	1B	70.3	67.5–73.2	Kukri_c18109_331–1104505	636.3–647.6	1	AUDPC
MQTL1B.3	1B	79.4	78.5–80.4	wsnp_RFL_Contig3951_4390396–1212781	646.2–661.6	1	AUDPC
MQTL1B.4	1B	91.2	90.6–91.8	wsnp_Ra_c53181_56932563–1103042	664.8–670.6	6	AUDPC, LR
MQTL1B.5	1B	96.9	95.1–98.8	BS00000010_51–1103838	670.8–678.6	2	AUDPC, LTN
MQTL1B.6	1B	106.8	106.0–106.92	5971316–Tdurum_contig49509_606	681.0–689.0	5	IR, AUDPC, MDS
MQTL1D.1	1D	2.6	1.5–3.8	wsnp_Ex_c278_538285–TA003600‐0503	0.4–0.5	2	DS, IR
MQTL1D.2	1D	23.9	22.7–25.0	1697264–Xwmc336	1.8–10.7	5	DS, IR
MQTL1D.3	1D	50.8	48.3–53.3	Excalibur_rep_c75168_337–1004137	15.4–22.3	2	DS, AUDPC
MQTL1D.4	1D	76.1	72.8–79.3	1068907–BS00028216_51	377.9–418.3	1	DS
MQTL2A.1	2A	15.6	13.5–17.7	1092460–3938659	71.2–104.9	1	IR
MQTL2A.2	2A	36.0	33.2–38.8	2263051–BS00045171_51	7.7–15.9	6	IR, LP, DS, LS
MQTL2A.3	2A	89.9	89.7–90.1	1217255–4910857	729.9–752.3	4	LIA, DS, IR
MQTL2B.1	2B	50.9	50.2–51.6	IAAV8205–Excalibur_c1787_1301	10.8–16.8	4	HR, AUDPC, IR
MQTL2B.2	2B	61.0	59.7–62.4	BS00022298_51–wsnp_Ra_c4321_7860456	17.6–24.9	4	LIA, IT, MDS, AUDPC
MQTL2B.3	2B	72.7	69.4–76.0	Excalibur_c2454_333–Excalibur_c13413_1008	26.6–30.5	3	MDS, AUDPC, 1R
MQTL2B.4	2B	96.9	94.9–98.9	Tdurum_contig54704_176–BS00096182_51	58.3–65.4	5	AUDPC, LP
MQTL2B.5	2B	106.9	106.2–107.7	Ku_c34010_1016–1107259	154.7–176.2	4	IT, AUDPC, LR
MQTL2B.6	2B	114.0	111.7–116.4	Kukri_c35153_956–1187788	540.0–712.8	2	LR
MQTL2B.7	2B	128.1	126.2–130.1	1094755–1381445	737.2–765.3	2	AUDPC, LIA
MQTL2D.1	2D	2.7	1.0–4.4	IAAV298–D_contig74612_253	1.7–10.3	7	DS, LP
MQTL2D.2	2D	33.4	31.6–35.3	1090614–1059345	37.2–49.9	5	DS, SPS, LTN
MQTL2D.3	2D	50.6	48.8–52.4	D_GBB4FNX01DJHXL_88–1101216	50.9–75.7	1	DS
MQTL2D.4	2D	72.0	69.7–74.3	RAC875_c42714_775–1102692	461.3–565.0	4	DS
MQTL3A.1	3A	11.7	8.3–15.1	1334535–4394604	1.3–13.3	1	DS
MQTL3A.2	3A	23.9	21.5–26.4	1243077–wsnp_Ex_c8409_14170476	21.2–24.6	2	DS, AUDPC
MQTL3A.3	3A	70.8	70.6–71.0	RAC875_c583_341–IACX333	662.4–674.7	8	LIA, DS, IT, AUDPC
MQTL3B.1	3B	11.7	10.7–12.7	wsnp_Ex_c30368_39293103–1213030	3.4–6.4	3	LIA, IT
MQTL3B.2	3B	27.2	24.6–29.7	1230466–1121919	11.7–24.4	8	AUDPC, SPL, IT, LP, IR, IE, LS
MQTL3B.3	3B	70.1	69.3–71.0	3947488–1262486	580.5–615.8	5	AUDPC, IT, LIA
MQTL3D.1	3D	9.5	6.7–12.2	1228026–2244235	7.3–13.3	1	DS
MQTL3D.2	3D	21.3	11.8–30.9	3026734–Kukri_c5252_107	12.9–31.7	1	DS
MQTL4A.1	4A	27.1	21.6–32.7	4909758–3022955	56.3–91.7	2	IT, DS
MQTL4A.2	4A	38.6	35.5–41.6	3948059–3021557	108.8–177.5	3	LR, DS, IR
MQTL4A.3	4A	54.6	50.9–58.2	BobWhite_c12128_187–1387412	610.5–670.9	3	LR, DS
MQTL4B.1	4B	34.1	32.4–35.7	1105735–wsnp_Ex_c30695_39579408	14.3–20.6	5	DS, IR
MQTL4B.2	4B	50.5	48.6–52.4	1210331–Tdurum_contig4974_1386	53.0–95.7	5	DS
MQTL4B.3	4B	99.5	96.1–102.9	1211581–BS00016834_51	656.9–667.7	2	IR, DS
MQTL4D.1	4D	36.7	35.4–37.9	2261222–1201923	15.0–23.3	4	IT, LIA, AUDPC, DS
MQTL4D.2	4D	78.4	72.8–84.1	1314743–1130355	465.2–497.9	4	DS, AUDPC, LS, SPL
MQTL5A.1	5A	30.2	27.4–33.1	3064538–995502	26.8–34.7	1	LTN
MQTL5A.2	5A	41.3	39.1–43.5	BS00022110_51–wsnp_Ra_c17216_26044790	440.3–549.6	3	DS, IR
MQTL5A.3	5A	55.0	52.2–57.8	3025345–1215538	554.3–586.3	4	DS, AUDPC
MQTL5A.4	5A	130.7	124.1–137.4	Xwmc110–1084271	644.9–685.0	1	EA‐
MQTL5B.1	5B	18.9	15.2–22.6	wsnp_Ex_rep_c105478_89891634–3021398	485.9–531.2	3	AUDPC, LR
MQTL5B.2	5B	46.1	44.1–48.2	3023016–982446	609.1–657.4	1	IT
MQTL5B.3	5B	69.2	66.4–72.0	2293625–1060195	685.2–704.2	4	LIA, IT, LTN, AUDPC
MQTL5B.4	5B	142.1	135.9–148.4	Xwmc235–1280229	634.2–711.9	4	IT, LIA
MQTL5D.1	5D	8.15	0.0–25.2	1091611–2289853	1.6–3.5	1	DS
MQTL5D.2	5D	43.4	41.8–44.9	1038800–BobWhite_c29857_228	5.6–25.3	1	DS
MQTL6A.1	6A	15.5	13.3–17.8	Kukri_c38982_165–1112318	11.1–17.9	4	DS, LP
MQTL6A.2	6A	33.5	30.9–36.1	4909358–1238725	33.5–49.8	5	DS, AUDPC, LR
MQTL6A.3	6A	48.2	44.2–52.1	1237185–wsnp_Ku_c4296_7807837	447.4–520.7	2	DS, LR
MQTL6B.1	6B	6.7	6.3–7.1	1139996–GENE‐4226_445	4.6–9.0	2	DS, AUDPC
MQTL6B.2	6B	19.6	14.2–25.1	RAC875_c62256_577–wsnp_Ex_c4815_8597064	17.7–30.1	2	DS, AUDPC
MQTL6B.3	6B	44.	40.5–48.5	IAAV8279–wsnp_Ex_c19874_28891457	514.0–665.1	4	DS, AUDPC
MQTL6B.4	6B	71.3	70.3–72.2	1094048–1228581	701.4–708.1	2	DS, AUDPC
MQTL6B.5	6B	82.8	81.2–84.4	3949402–2275538	713.6–715.2	4	DS, AUDPC, LR
MQTL6B.6	6B	106.2	105.9–106.4	1231000–Tdurum_contig10729_64	717.9–721.0	2	DS, AUDPC
MQTL7A.1	7A	74.4	73.8–74.9	1099858–BS00047811_51	69.7–79.6	1	LR
MQTL7A.2	7A	86.9	80.0–93.9	7345647–IACX2471	554.4–669.7	1	LR
MQTL7A.3	7A	117.0	107.7–126.3	Tdurum_contig29834_165–1002615	679.9–714.6	1	DS
MQTL7A.4	7A	130.2	128.9–131.4	3956725–wPt‐0745	716.4–736.2	5	DS, AUDPC
MQTL7B.1	7B	74.9	73.0–76.8	Tdurum_contig47317_100–wsnp_Ex_c19_39269	675.1–693.3	7	IR, LR, AUDPC, IT, LTN
MQTL7B.2	7B	93.5	92.3–94.7	1114524–BobWhite_c2892_167	700.7–721.2	3	LR, LP, AUDPC
MQTL7B.3	7B	122.3	120.4–124.3	wPt‐2933–Xwmc10	734.3–744.9	3	LR, LTN, IR
MQTL7B.4	7B	141.7	141.7–141.7	Xbarc32–RAC875_c525_1372	723.9–750.1	9	EA‐, EA+, EST‐, LIA, EST+, NEC, LP, AUDPC
MQTL7D.1	7D	25.8	25.4–26.2	D_contig24676_377–1103360	167.6–185.1	8	LTN, DS, LR, AUDPC, LS
MQTL7D.2	7D	42.4	41.2–43.6	2246156–2244056	569.2–591.7	4	MDS, AUDPC, DS
MQTL7D.3	7D	58.5	57.9–59.1	1242088–1123852	610.9–629.7	4	AUDPC, DS, LTN
MQTL7D.4	7D	148.6	137.7–159.5	3533423–wPt‐666144	636.3–638.2	1	LTN

^a^Chr, chromosome.

^b^CI, confidence interval.

^c^DS, disease severity; AUDPC, area under the disease progress curve; LIA, leaf infected area; IT, infection type; IR, infection rate; LR, leaf rust resistance; LTN, leaf tip necrosis, LP, latent period; SPS, spore production per unit of sporulating tissue; SPL, spore production per lesion; IE, infection efficiency; LS, lesion size; EA−, early aborted colonies, associated with plant cell necrosis; EA+, early aborted colonies, without plant cell necrosis; EST−, established colonies, associated with plant cell necrosis; EST+, established colonies, without plant cell necrosis; NEC, all necrotic colonies; MDS, maximum disease severity; HR, host reaction.

### 3.3 Association study validation and colocalization with leaf rust genes

**FIGURE 2:**
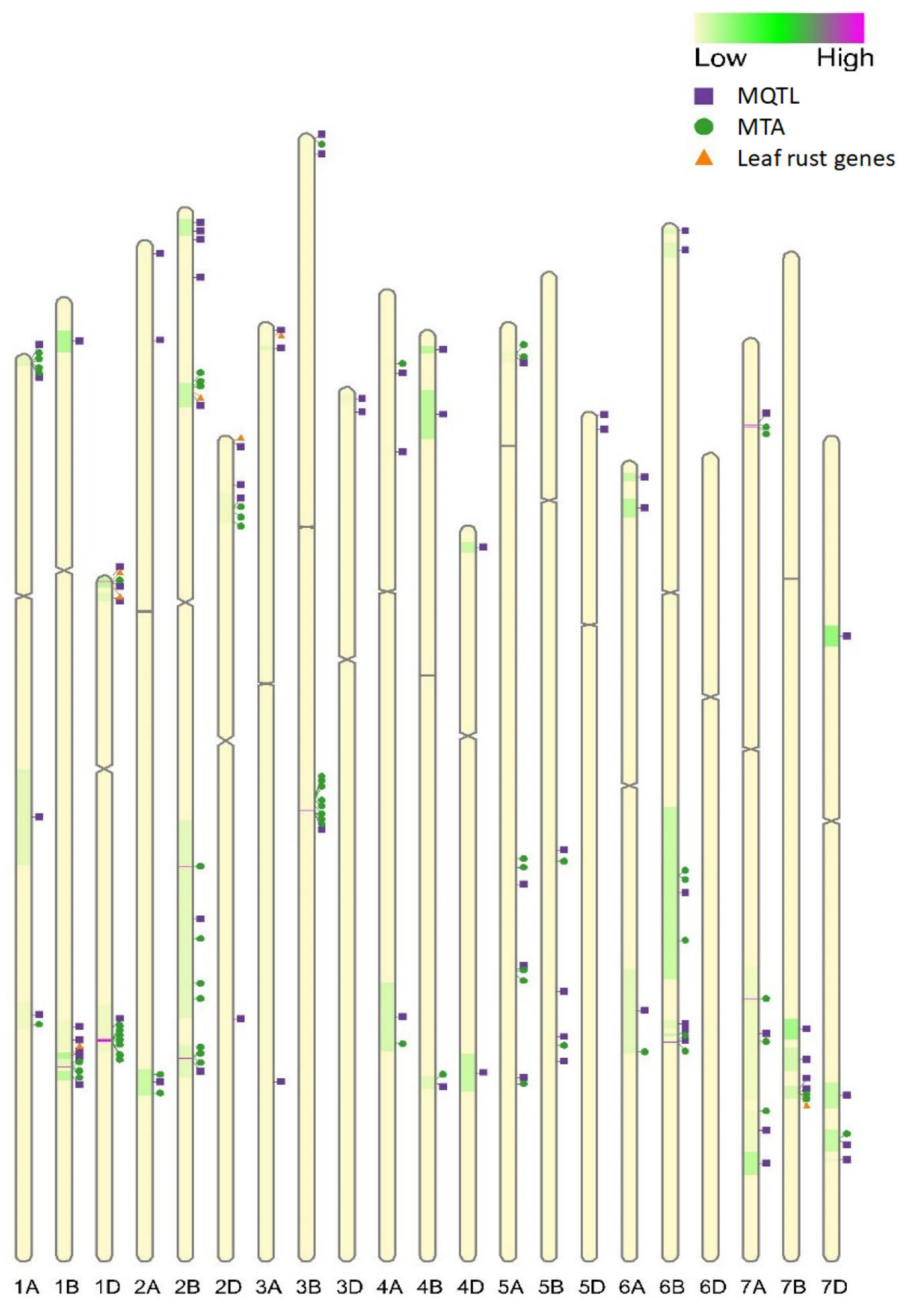
Distribution of the meta‐quantitative trait loci (MQTL), marker–trait associations (MTAs), and colocalized leaf rust genes with reference to the Chinese Spring genome. The chromosome coloration correlates to the number of initial QTL, the lighter color connotes less QTL. The centromere of each chromosome is represented by the constriction

The loci resistant to leaf rust across different genetic backgrounds and environments identified in recent association studies (Supplemental Table S3) were used to validate the MQTL discovered in this study. A total of 51 marker–trait associations identified were colocalized with 29 MQTL; thus, some MQTL integrated more than one marker–trait association (Supplemental Table S4; Figure 2). Furthermore, eight leaf rust genes colocalized with some MQTL found in this study (Supplemental Table S5; Figure 2), such as MQTL1D.2 colocalizing with *Lr60* and *Lr42* and MQTL7B.3 colocalizing with the *Lr68* and *Lr14a* genes. Additionally, MQTL1B.4 and MQTL2B.5 colocalized with two leaf rust genes, *Lr46* and *Lr13*, respectively, conferring adult plant resistance.

### 3.4 Candidate gene mining of established hcmQTL

To further improve the quality of the MQTL discovered, they were further refined to regions termed hcmQTL. The hcmQTL consist of 15 consensus regions (Table 2; Supplemental Table S6), having an average CI and physical interval of 2.9 cM and 12.04 Mb, respectively. Overall, each hcmQTL cluster contained at least five QTL. The B genome had the highest number of these hcmQTL (7). Within the B genome, chromosome 1B contained the highest number of hcmQTL (3). In addition, hcmQTL4B.1 had the smallest physical interval (6.25 Mb), while hcmQTL7A.4 had the largest interval, covering 19.78 Mb. Afterward, candidate gene mining within hcmQTL revealed 2,240 genes, with hcmQTL7A.4 possessing the highest number of candidate genes and hcmQTL2B.4 possessing the lowest number (18) of candidate genes.

**TABLE 2:** The high‐confidence meta‐quantitative trait loci (hcmQTL) for leaf rust resistance detected in this study. It is indicated the position (Pos) of the QTL in the genetic map and the confidence intervals (CI) in the genetic map and genome sequence

**hcmQTL**	**Chr** **^a^**	**Pos**	**CI (cM)**	**Flanking markers**	**CI**	**No. QTL**	**Traits** **^b^**	**Resistance source**	**No. candidate genes mined**
		cM			Mb				
hcmQTL1B.1	1B	36.4	35.4–37.3	1770278–1108935	29.6–48.1	6	LR, LIA, IT, AUDPC	Capo, Forno, MG5323, Sujata	126
hcmQTL1B.4	1B	91.2	90.6–91.8	wsnp_Ra_c53181_56932563–1103042	664.8–670.6	6	AUDPC, LR	Bairds, Chileno, Dunkler, Kundan	114
hcmQTL1B.6	1B	106.8	106.6–106.9	5971316–Tdurum_contig49509_606	681.0‐ 689.0	5	IR, AUDPC, MDS	Francolin#1, W014204	81
hcmQTL1D.2	1D	23.9	22.7–25.0	1697264–Xwmc336	1.8–10.7	5	DS, IR	Vesper	228
hcmQTL2A.2	2A	36.0	33.2–38.8	2263051–BS00045171_51	7.7–15.9	6	IR, LP, DS, LS	AC Cadillac, Carberry, Creso	204
hcmQTL2B.4	2B	96.9	94.9–98.9	Tdurum_contig54704_176–BS00096182_51	58.3–65.4	5	AUDPC, LP	Bairds, Dunkler, Kariega	18
hcmQTL2D.1	2D	2.7	1.0–4.4	IAAV298–D_contig74612_253	1.7–10.3	7	DS, LP	C615, CI13227, Fundulea 900, Kundan, MN99394‐1, SHA/CBRD	204
hcmQTL2D.2	2D	33.5	31.6–35.3	1090614–1059345	37.2–49.9	5	DS, SPS, LTN	Apache, Forno	77
hcmQTL3A.3	3A	70.8	70.6–71.0	RAC875_c583_341–IACX333	662.4–674.7	8	LIA, DS, IT, AUDPC	CDC Go, Lloyd, TA4152‐60, Zhou 8425B	123
hcmQTL3B.2	3B	27.2	24.6–29.7	1230466–1121919	11.7–24.4	8	AUDPC, SPL, IT, LP, IR, IE, LS	AC Cadillac, AC Taber, Apache, Balance CI13227, Opata 85	211
hcmQTL4B.1	4B	34.1	32.4–35.7	1105735–wsnp_Ex_c30695_39579408	14.3–20.6	5	DS, IR	Carberry, Saar	67
hcmQTL6A.2	6A	33.5	30.9–36.1	4909358–1238725	33.5–49.8	5	DS, AUDPC, LR	Apache, Paragon	181
hcmQTL7A.4	7A	130.2	128.9–131.4	3956725–wPt‐0745	716.4–736.2	5	DS, AUDPC	Apache	282
hcmQTL7B.1	7B	74.9	73.0–76.8	Tdurum_contig47317_100–wsnp_Ex_c19_39269	675.1–693.3	7	IR, LR, AUDPC, IT, LTN	Kenya Kongoni, MG5323, Oberkulmer, Red Fife	174
hcmQTL7D.1	7D	25.8	25.4–26.2	D_contig24676_377–1103360	167.6–185.1	8	LTN, DS, LR, AUDPC, LS	Carberry, Chapio, Fundulea 900, Saar	150

^a^Chr, chromosome.

^b^DS, disease severity; AUDPC, area under the disease progress curve; LIA, leaf infected area; IT, infection type; IR, infection rate; LR, leaf rust resistance; LTN, leaf tip necrosis, LP, latent period; SPS, spore production per unit of sporulating tissue; SPL, spore production per lesion; IE, infection efficiency; LS, lesion size; EA−, early aborted colonies, associated with plant cell necrosis; EA+, early aborted colonies, without plant cell necrosis; EST−, established colonies, associated with plant cell necrosis; EST+, established colonies, without plant cell necrosis; NEC, all necrotic colonies; MDS, maximum disease severity; HR, host reaction.

### 3.5 Discovering DEGs within hcmQTL

**FIGURE 3:**
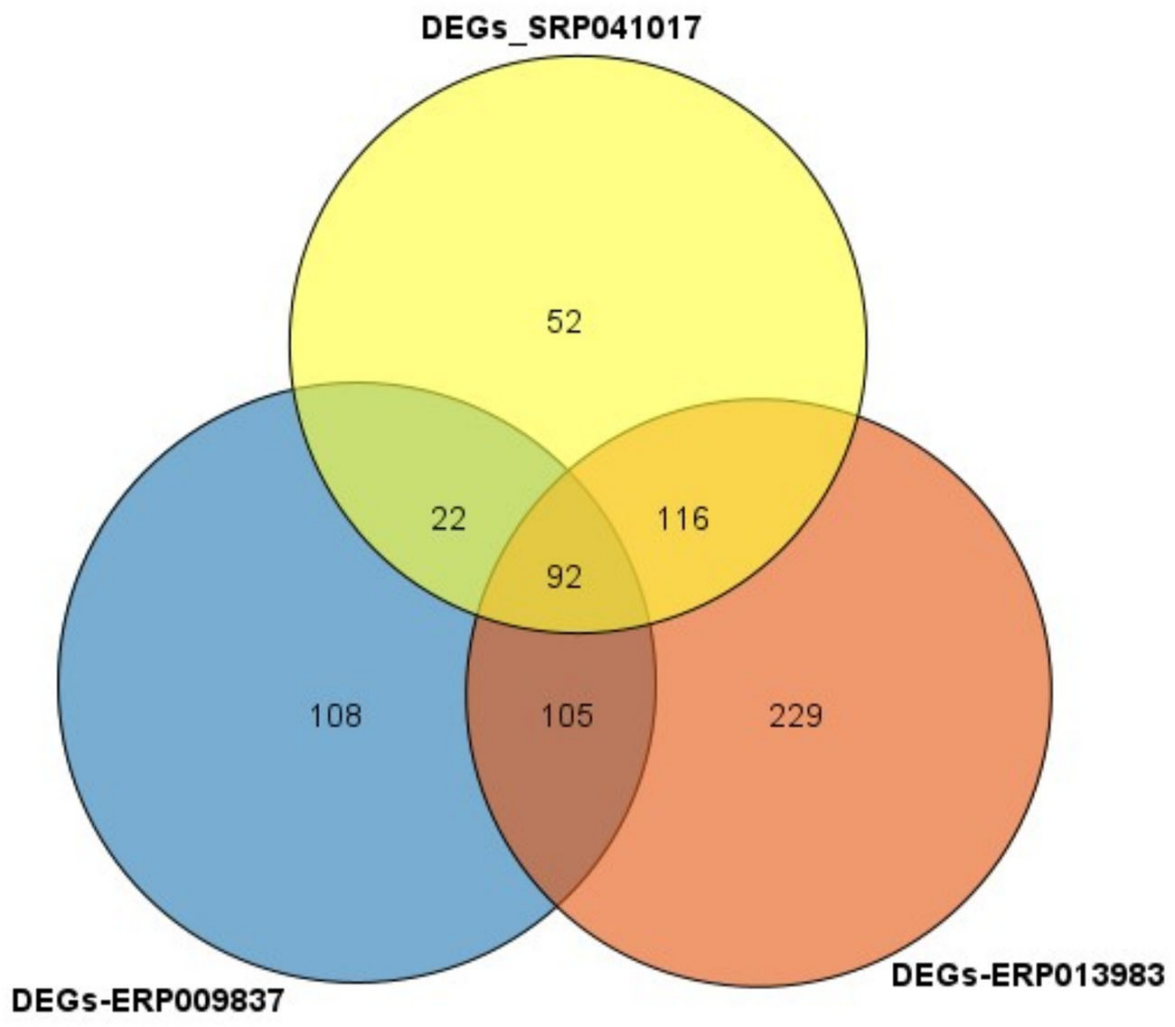
Venn diagram depicting the number of differentially expressed genes (DEGs) from three transcriptomic data sets. The Venn diagram visually illustrates the number of DEGs that were identified in three transcriptomic data sets: ERP013983, SRP041017, and ERP009837

**FIGURE 4:**
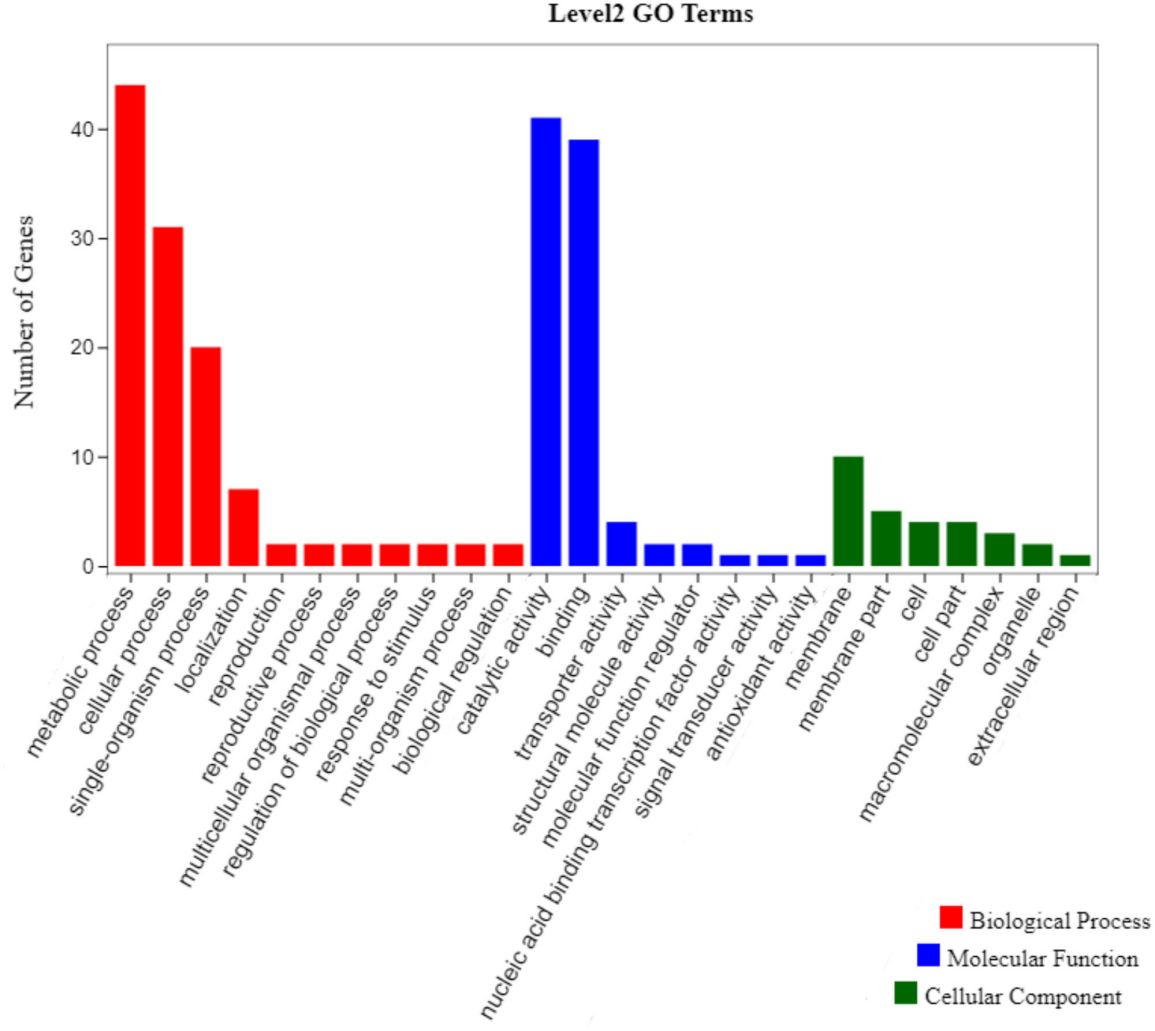
Level 2 gene ontology (GO) terms for differentially expressed genes (DEGs) in the hcmQTL regions

Owing to the lack of transcriptomic data repositories for leaf rust in wheat, we decided to use transcriptomic expression data for three fungal diseases in wheat—stripe rust, powdery mildew, and *Septoria tritici* blotch—to explore the DEGs within these hcmQTL. The ERP013983 dataset revealed 541 DEGs with 221 downregulated genes, 255 upregulated genes, and 65 genes that were downregulated under several conditions and upregulated under others (Supplemental Table S7; Figure 3). From this dataset, hcmQTL7A.4 had the highest number of DEGs (59), while hcmQTL2B.4 had the lowest (12). The SRP041017 dataset revealed 289 DEGs, where 131 genes were downregulated, 154 genes were upregulated, and four genes were downregulated at one time point and upregulated in others (Supplemental Table S8; Figure 3). From this dataset, hcmQTL2A.2 had the highest number of DEGs (39), while hcmQTL2B.4 and hcmQTL4B.1 had the fewest DEGs (4). From the third dataset, ERP009837, a total of 327 DEGs were discovered, with 125 genes downregulated, 198 genes upregulated, and four genes downregulated at one time point and upregulated in others (Supplemental Table S9; Figure 3). From this dataset, hcmQTL2A.2 had the highest number of DEGs (34), while hcmQTL2B.4 had the lowest number of DEGs (3). A total of 92 genes were found to be differentially expressed across the three expression datasets used. The 92 DEGs within the hcmQTL interval were analyzed for GO enrichment (Table 3). The most significantly enriched GO terms associated with biological processes were for metabolic (44 genes) and cellular processes (31 genes) (Supplemental Table S10; Figure 4). The most significantly enriched GO terms associated with molecular function were for catalytic activities (41 genes) and binding (39 genes). In terms of cellular components, the genes were enriched mainly in the cell membrane and its components.

**TABLE 3:** High‐confidence meta‐quantitative trait loci (hcmQTL) that showed differential expression across the three transcriptomics datasets

**Candidate gene**	**hcmQTL**	**Chr**	**Start**	**End**	**Functional annotation**
			bp		
TraesCS1B02G062400.1	hcmQTL1B.1	1B	47,205,580	47,206,723	Polyadenylate‐binding protein 1‐B‐binding protein
TraesCS1B02G059900.1	hcmQTL1B.1	1B	41,366,476	41,367,409	Protein trichome birefringence
TraesCS1B02G451100.1	hcmQTL1B.4	1B	668,911,604	668,912,857	Chlorophyll a‐b binding protein, chloroplastic
TraesCS1B02G455200.1	hcmQTL1B.4	1B	670,384,230	670,390,259	ATP‐dependent zinc metalloprotease FtsH 1
TraesCS1B02G454600.1	hcmQTL1B.4	1B	670,164,777	670,167,783	Receptor‐like protein kinase, putative, expressed
TraesCS1B02G452000.1	hcmQTL1B.4	1B	669,027,344	669,033,812	CAS1 domain‐containing protein 1
TraesCS1B02G454100.1	hcmQTL1B.4	1B	670,034,245	670,037,207	Receptor‐like protein kinase, putative, expressed
TraesCS1B02G452100.1	hcmQTL1B.4	1B	669,150,552	669,154,353	Solanesyl diphosphate synthase family protein
TraesCS1B02G448300.1	hcmQTL1B.4	1B	667,677,457	667,682,364	Sugar transporter protein
TraesCS1B02G452200.1	hcmQTL1B.4	1B	669,157,668	669,163,604	Mitogen‐activated protein kinase
TraesCS1B02G451200.1	hcmQTL1B.4	1B	668,911,879	668,912,613	phosphatidylinositol‐4‐phosphate 5‐kinase 2
TraesCS1B02G454400.1	hcmQTL1B.4	1B	670,152,915	670,155,867	Receptor‐like protein kinase, putative, expressed
TraesCS1B02G479800.1	hcmQTL1B.6	1B	687,407,942	687,418,205	Structural maintenance of chromosomes
TraesCS1D02G013800.1	hcmQTL1D.2	1D	6,898,428	6,901,098	Serine/threonine‐protein kinase
TraesCS1D02G004600.1	hcmQTL1D.2	1D	2,124,693	2,127,355	Cytochrome P450
TraesCS1D02G013200.1	hcmQTL1D.2	1D	6,774,424	6,778,290	Ras‐like protein
TraesCS1D02G003700.1	hcmQTL1D.2	1D	2,066,324	2,067,459	50S ribosomal protein L28
TraesCS1D02G004700.1	hcmQTL1D.2	1D	2,139,321	2,145,297	Phospholipid‐transporting ATPase
TraesCS1D02G009100.1	hcmQTL1D.2	1D	4,821,093	4,828,103	Glucan 1,3‐beta‐glucosidase
TraesCS1D02G018700.1	hcmQTL1D.2	1D	8,182,627	8,186,066	NBS‐LRR disease resistance protein
TraesCS1D02G020600.1	hcmQTL1D.2	1D	8,836,433	8,837,400	Trypsin inhibitor
TraesCS1D02G017700.1	hcmQTL1D.2	1D	7,874,518	7,876,881	Receptor‐like kinase
TraesCS2A02G031400.1	hcmQTL2A.2	2A	14,468,158	14,485,592	NBS‐LRR‐like resistance protein
TraesCS2A02G026800.1	hcmQTL2A.2	2A	12,315,023	12,316,569	2‐OG and Fe(II)‐dependent oxygenase superfamily
TraesCS2A02G027600.1	hcmQTL2A.2	2A	12,737,989	12,742,737	Kaurene synthase
TraesCS2A02G031500.1	hcmQTL2A.2	2A	14,529,282	14,533,746	NBS‐LRR‐like resistance protein
TraesCS2A02G026900.1	hcmQTL2A.2	2A	12,324,869	12,327,912	HR‐like lesion‐inducing protein‐related protein
TraesCS2A02G027500.1	hcmQTL2A.2	2A	12,698,384	12,702,381	Cytochrome P450
TraesCS2A02G024700.1	hcmQTL2A.2	2A	11,909,066	11,911,836	Receptor‐like protein kinase
TraesCS2A02G027800.1	hcmQTL2A.2	2A	12,893,128	12,897,700	Copalyl diphosphate synthase
TraesCS2A02G022700.1	hcmQTL2A.2	2A	10,992,541	10,996,031	ATP‐dependent Clp protease adapter protein clpS
TraesCS2A02G028700.1	hcmQTL2A.2	2A	13,013,184	13,015,074	Glycosyltransferase
TraesCS2A02G028000.1	hcmQTL2A.2	2A	12,911,511	12,913,256	Glycosyltransferase
TraesCS2A02G035100.1	hcmQTL2A.2	2A	15,269,852	15,270,683	Glyceraldehyde‐3‐phosphate dehydrogenase
TraesCS2A02G030700.1	hcmQTL2A.2	2A	14,069,433	14,071,940	Exocyst complex component, putative
TraesCS2B02G101700.1	hcmQTL2B.4	2B	62,549,905	62,556,194	AMP deaminase
TraesCS2B02G104200.1	hcmQTL2B.4	2B	64,987,460	64,990,527	Protein kinase family protein, putative, expressed
TraesCS2D02G008200.1	hcmQTL2D.1	2D	4,458,207	4,460,513	ATP synthase E chain
TraesCS2D02G017400.1	hcmQTL2D.1	2D	8,345,960	8,348,996	Serine/threonine‐protein kinase
TraesCS2D02G021300.1	hcmQTL2D.1	2D	9,873,285	9,874,766	Glycosyltransferase
TraesCS2D02G013300.1	hcmQTL2D.1	2D	6,599,740	6,603,424	Receptor‐kinase, putative
TraesCS2D02G011700.1	hcmQTL2D.1	2D	5,668,870	5,674,210	WRKY transcription factor
TraesCS2D02G010100.1	hcmQTL2D.1	2D	5,191,027	5,192,702	NBS‐LRR‐like resistance protein
TraesCS2D02G017500.1	hcmQTL2D.1	2D	8,377,694	8,379,215	Protein kinase
TraesCS2D02G021500.1	hcmQTL2D.1	2D	9,882,737	9,884,634	Cytochrome P450
TraesCS2D02G003200.1	hcmQTL2D.1	2D	2,257,606	2,263,626	NBS‐LRR‐like resistance protein
TraesCS2D02G016900.1	hcmQTL2D.1	2D	8,281,359	8,289,277	LIM domain‐containing protein 1
TraesCS2D02G088600.1	hcmQTL2D.2	2D	38,543,206	38,546,253	Leucine‐rich repeat receptor‐like protein kinase
TraesCS2D02G093100.1	hcmQTL2D.2	2D	44,468,558	44,470,302	Hexosyltransferase
TraesCS2D02G088500.1	hcmQTL2D.2	2D	38,250,501	38,252,827	Lectin receptor kinase
TraesCS3A02G432700.1	hcmQTL3A.3	3A	674,023,677	674,025,582	Tetratricopeptide repeat (TPR)‐like superfamily
TraesCS3A02G422100.1	hcmQTL3A.3	3A	663,435,887	663,439,805	Selenium‐binding protein
TraesCS3A02G423600.1	hcmQTL3A.3	3A	665,980,620	665,987,221	Leucine‐rich repeat receptor‐like protein kinase
TraesCS3A02G428300.1	hcmQTL3A.3	3A	671,718,886	671,724,129	Glycosyltransferase
TraesCS3A02G426700.1	hcmQTL3A.3	3A	669,619,467	669,624,388	Auxin efflux carrier component
TraesCS3B02G030600.1	hcmQTL3B.2	3B	13,960,570	13,967,611	Receptor‐like kinase
TraesCS3B02G039700.1	hcmQTL3B.2	3B	19,257,562	19,261,346	Nuclease S1
TraesCS3B02G039900.1	hcmQTL3B.2	3B	19,263,438	19,270,330	Transmembrane protein 214
TraesCS3B02G047300.1	hcmQTL3B.2	3B	23,957,233	23,962,495	Hexose transporter
TraesCS3B02G034600.1	hcmQTL3B.2	3B	16,457,625	16,463,204	Cellulose synthase family protein, expressed
TraesCS3B02G041900.1	hcmQTL3B.2	3B	20,840,849	20,842,894	Tryptophan synthase alpha chain
TraesCS4B02G025500.1	hcmQTL4B.1	4B	18,162,017	18,167,541	Homeobox protein BEL1 like
TraesCS6A02G071000.1	hcmQTL6A.2	6A	38,758,958	38,759,492	Inhibitor protein
TraesCS6A02G078400.1	hcmQTL6A.2	6A	48,193,117	48,193,962	Plant basic secretory family protein
TraesCS6A02G072500.1	hcmQTL6A.2	6A	39,495,492	39,505,834	aminoalcoholphosphotransferase 1
TraesCS6A02G073300.1	hcmQTL6A.2	6A	40,217,272	40,222,244	3‐ketoacyl‐CoA synthase
TraesCS6A02G076200.1	hcmQTL6A.2	6A	46,696,621	46,700,587	Ubiquitin thioesterase
TraesCS6A02G076300.1	hcmQTL6A.2	6A	46,702,941	46,706,410	NADH‐ubiquinone oxidoreductase subunit
TraesCS6A02G077000.1	hcmQTL6A.2	6A	47,104,969	47,106,326	Xylanase inhibitor protein 1
TraesCS7A02G553000.1	hcmQTL7A.4	7A	725,912,026	725,915,959	Aquaporin
TraesCS7A02G558500.1	hcmQTL7A.4	7A	730,749,198	730,749,974	Thaumatin‐like protein
TraesCS7A02G560400.1	hcmQTL7A.4	7A	732,087,221	732,090,195	Peptidoglycan‐binding LysM domain protein
TraesCS7A02G545200.1	hcmQTL7A.4	7A	721,222,769	721,223,586	Thiosulfate sulfurtransferase GlpE
TraesCS7A02G552400.1	hcmQTL7A.4	7A	725,433,279	725,435,212	High mobility group protein
TraesCS7A02G549800.1	hcmQTL7A.4	7A	724,082,577	724,090,076	Catalase
TraesCS7A02G560200.1	hcmQTL7A.4	7A	732,052,718	732,055,355	Photosystem II stability/assembly factor HCF136
TraesCS7A02G544700.1	hcmQTL7A.4	7A	721,093,026	721,106,211	DNA helicase
TraesCS7B02G408200.1	hcmQTL7B.1	7B	677,449,647	677,453,105	Tetraspanin family protein
TraesCS7B02G412700.1	hcmQTL7B.1	7B	680,276,900	680,281,365	Anthocyanidin reductase
TraesCS7B02G423900.1	hcmQTL7B.1	7B	693,190,704	693,194,113	Indole‐3‐glycerol phosphate synthase‐like
TraesCS7B02G411800.1	hcmQTL7B.1	7B	680,095,492	680,097,691	Patatin
TraesCS7B02G420700.1	hcmQTL7B.1	7B	690,012,782	690,017,154	Lysosomal Pro‐X carboxypeptidase
TraesCS7B02G415000.1	hcmQTL7B.1	7B	682,885,762	682,886,701	Chaperone protein dnaJ
TraesCS7B02G417700.1	hcmQTL7B.1	7B	685,478,267	685,479,217	Thaumatin‐like protein
TraesCS7B02G421100.1	hcmQTL7B.1	7B	690,238,969	690,240,738	50S ribosomal protein L35
TraesCS7D02G220300.1	hcmQTL7D.1	7D	180,685,905	180,691,434	CONSTANS‐like zinc finger protein
TraesCS7D02G211000.1	hcmQTL7D.1	7D	169,382,246	169,386,423	Aspartic proteinase Asp1
TraesCS7D02G212800.1	hcmQTL7D.1	7D	171,302,855	171,306,748	Receptor‐like kinase
TraesCS7D02G222200.1	hcmQTL7D.1	7D	183,327,364	183,330,163	Protein FATTY ACID EXPORT 1, chloroplastic
TraesCS7D02G214900.1	hcmQTL7D.1	7D	174,441,593	174,445,075	Kinase family protein
TraesCS7D02G217700.1	hcmQTL7D.1	7D	178,291,737	178,293,161	Glycosyltransferase
TraesCS7D02G212700.1	hcmQTL7D.1	7D	170,957,942	170,970,018	Importin, putative

*Note*. Chr, Chromosomes

### 3.6 Tissue‐specific expression profile of DEGs within hcmQTL

**FIGURE 5:**
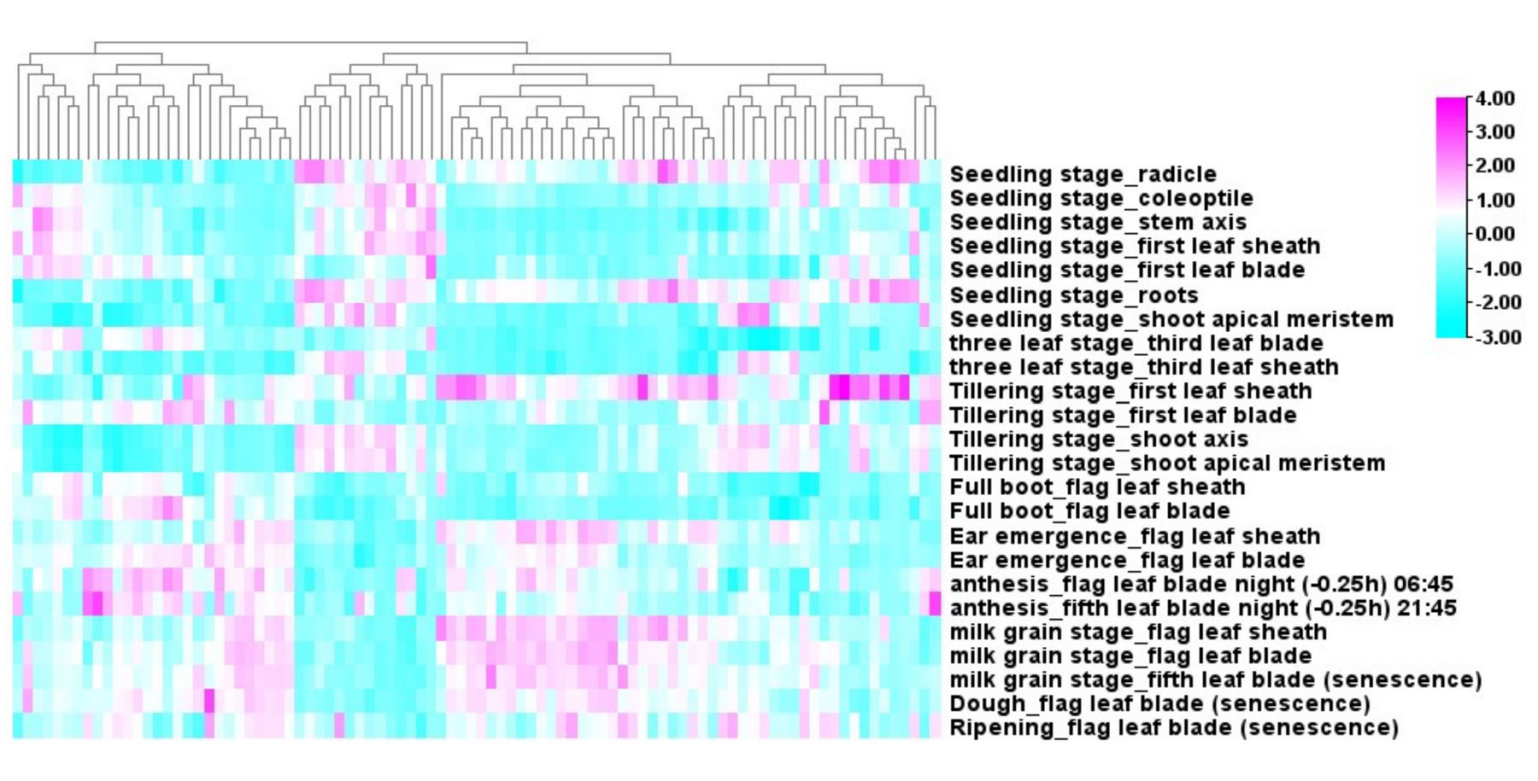
Expression pattern of 90 candidate genes in 24 tissues. All transcriptome data was downloaded from expVIP (http://www.wheat‐expression.com). The changes in color from sky blue to pink signifies alteration in level of expression from low to high

To analyze the differential expression within the hcmQTL at different tissues and development stages, three transcriptomics datasets were used: SRP041017, ERP013983, and ERP009837. Row clustering was applied, and, as a result, the 92 DEGs fell into two classes based on their expression patterns (Figure 5). Genes in Class I showed moderate‐to‐high expression in the flag leaf blade and fifth leaf blade at the anthesis stage when compared with other stages of growth. Moreover, for Class II, more genes were highly expressed in the first leaf sheath at the tillering stage. The DEGs in Class II accounted for more than half of the overall DEGs, and they showed contrasting expression patterns to those shown by genes in Class I. For Class I, at the seedling stage, the genes *TraesCS1D02G003700* (hcmQTL1D.2) and *TraesCS7B02G421100* (hcmQTL7B.1) showed moderate expression in the stem axis, while at the adult stage, *TraesCS6A02G07250*0 (hcmQTL6A.2) was highly expressed in the fifth leaf blade at the anthesis stage, and *TraesCS1D02G017700* (hcmQTL1D.2) showed high expression in the flag leaf at the dough stage. Furthermore, at the seedling stage, more Class II genes were moderately expressed in the radicle and roots, with only T*raesCS7D02G21100*0 (hcmQTL7D.1) showing expression in the shoot apical meristem. At the adult stage, the gene *TraesCS7D02G220300* (hcmQTL7D.1) was highly expressed in the fifth leaf blade at the anthesis stage.

## 4 DISCUSSION

### 4.1 Establishment of MQTL regions

To gain deeper insight into the control of leaf rust resistance in wheat, an MQTL analysis was performed based on the numerous QTL conferring leaf rust resistance identified in the literature from various independent studies. The first step to identifying consensus regions via MQTL analysis is the projection of the original QTL onto a consensus or reference map.

A feature of the consensus map and QTL database was that the B genome reported the highest marker saturation, and thus, the highest number of QTL was mapped to this genome, which is in agreement with previous studies characterizing genetic diversity and unravelling complex traits for disease resistance in bread wheat (Soriano & Royo, 2015; Wang et al., 2014). The D genome presented a lower number of QTL, as previously found in other MQTL analyses for disease resistance in wheat (Liu et al., 2020; Soriano & Royo, 2015; Venske et al., 2019; Zheng et al., 2020). Furthermore, no QTL were found on chromosome 6D, as discovered in previous MQTL analysis studies on leaf rust and Fusarium head blight diseases in wheat (Soriano & Royo, 2015; Zheng et al., 2020). A possible explanation for the limited QTL located on the D genome across various disease studies could be the low level of polymorphism associated with the D genome. In this study, a larger number (81.4%) of QTL was projected onto the consensus map compared with the fewer number of QTL projected in a previous MQTL analysis for leaf rust (Soriano & Royo, 2015) (44%). A possible reason could be due to the different consensus maps used. In this study, we used a high‐density consensus map that combined SSRs and markers obtained from high‐throughput genotyping platforms, in contrast to the consensus map used in the previous study from Soriano and Royo (2015). Consequently, the number of consensus genomic regions (MQTL) discovered in this study was higher than those reported in Soriano and Royo (2015), at 75 and 48, respectively. For the MQTL discovered in CI interval of the original QTL, ranging from 1.14 to 173.11 cM. In addition, in the present study, the physical position of the MQTL was reported because of the release of the wheat genome sequence (The International Wheat Genome Sequencing Consortium et al., 2018), improving the mapping resolution of the genome regions and helping the identification of candidate genes. These analyses enhance the results provided by studies published prior to the release of the genome sequence. In this study, we discovered seven MQTL incorporating at least five original QTL and having a confidence interval of <10 Mb, making them the most promising for candidate gene identification.

### 4.2 Colocalization of MQTL with leaf rust resistance genes and traits

To strengthen the location of MQTL discovered in this study, a search for colocalization of leaf rust resistance genes and MQTL was performed. More than 61 leaf rust genes have been mapped and documented in wheat (Kim et al., 2020), and four of them have been cloned (Hafeez et al., 2021). A total of six leaf rust genes (*Lr13, Lr14a, Lr46, Lr68, Lr63, Lr60, Lr42*, and *Lr41*) were found to colocalize with MQTL. Interestingly, the colocalization of *Lr13*, *Lr14a*, and *Lr46* with MQTL2B.5, MQTL7B.3, and MQTL1B.4 on chromosomes 2B, 7B, and 1B, respectively, in this study was in agreement with the results obtained by Soriano and Royo (2015). As reported by these authors, *Lr68* was found to have a tight association with MQTL33 (colocalized with *Lr14a*) on chromosome 7B; however, in this study, MQTL7B.3 colocalized with both leaf rust genes (*Lr14a* and *Lr68*), thus confirming the usefulness of using highly saturated consensus maps for meta‐QTL analysis. The gene *Lr14a*, known to confer seedling resistance, is thought to have evolved from emmer wheat [*Triticum turgidum* L. subsp. *dicoccon* (Schrank) Thell.] ‘Yaroslav’ (McFadden, 1930) and is associated with the stem rust and powdery mildew resistance genes *Sr17* and *Pm5*. *Lr68*, on the other hand, confers adult plant resistance to the majority of *P. triticina* isolates with low‐to‐medium infection types and is linked to small but noticeable leaf tip necrosis (Herrera‐Foessel et al., 2012). Consequently, the MQTL7B.3 region not only confers seedling and adult plant resistance to leaf rust but also constitutes a region of multiple disease resistance. Additionally, MQTL1D.2 colocalized with two leaf rust resistance genes (*Lr60* and *Lr42*). Hiebert et al. (2008) found that *Lr60* is 13.5 cM distal to *Lr21*, which would position *Lr60* and *Lr42* approximately 40 cM apart (Huang et al., 2003; Somers et al., 2004). The association between *Lr60* and *Lr42* has not been confirmed, but in this study, we discovered that both genes were located in the same MQTL, with a CI of 8.8 Mb. This supports possible linkage between the two genes; however, a genetic linkage test needs to be carried out to corroborate this claim. Furthermore, *Lr60* is known to confer seedling resistance, while *Lr42* confers adult plant resistance. Additionally, MQTL1B.4 colocalized with *Lr46*, a gene known to increase the latent period and reduce the frequency of infection and uredinial size in a similar manner to *Lr34* (Drijepondt & Pretorius, 1989; William et al., 2003). There is also a tight linkage between *Lr46* and a stripe rust gene (*Yr29)*, which is similar to the linkage between *Lr34* and *Yr18* (McIntosh, 1992; Singh, 1992). Consequently, the MQTL1D.2 region confers resistance to both leaf and stripe rust in wheat, thus making this region a hotspot for selecting multiple disease resistance in wheat.

Most of the MQTL discovered in this study clustered QTL conferring two or more resistance traits. In another study, Ren et al. (2012) also discovered that maximum disease severity had a significant association with the AUDPC across diverse environments, and this finding was in agreement with previous studies (Lan et al., 2009; Liang et al., 2006; Wang et al., 2005). Consequently, this result indicates the possibility of replacing AUDPC with MDS. A possible explanation for this could be that when two or more traits are mapped to the same region, they are most likely under the same genetic control, as suggested by Lu et al. (2017). Furthermore, effects arising from tight linkage and pleiotropism could also be a possible explanation.

### 4.3 Candidate genes within hcmQTL and their role in leaf rust resistance

The search for candidate genes was extended to hcmQTL within 20 Mb, thus yielding 15 hcmQTL. The hcmQTL also have a small CI compared with MQTL, thus making them more reliable and useful for QTL selection in breeding programs. Gene annotation of the hcmQTL identified a total of 2,240 genes, which were narrowed down to 92 DEGs after in silico transcriptomic analysis. Two main types of disease resistance are used in breeding programs: seedling resistance and adult plant resistance. Thus, the analysis of the expression of the candidate genes across different tissues and developmental stages can inform us of their potential role in seedling or adult plant resistance. Five out of the 92 genes expressed across the three transcriptomic data sets—*TraesCS7D02G212800*, *TraesCS6A0G073300*, *TraesCS2B02G104200*, *TraesCS1D02G003700*, and *TraesCS2D02G021300*—showed moderate expression in the first leaf sheath at the seedling stage. *TraesCS7D02G212800* and *TraesCS2B02G104200* encode a receptor‐like kinase (RLK) and protein kinase family protein, respectively, and both proteins play a crucial role in contributing to disease resistance in wheat. Plant protein kinases, as well as RLKs, govern the detection and activation of diverse developmental and physiological signals, particularly those involved in defense and symbiosis (Rentel et al., 2004; Abu‐Qamar et al. et al., 2008; Fu et al. et al., 2009; Garcia et al. et al., 2012). Prior studies found that various RLK genes coding wheat leaf rust kinases were conserved in wheat, with the most studied member of the wheat leaf rust kinase family being LRK10, which is genetically linked to the *Lr10* locus (Feuillet et al., 1998, 1997, 2001). Gu et al. (2020), in a recent study, uncovered an RLK gene that plays an important role in resistance to *P. triticina* infection and has a positive regulatory effect on the hypersensitive reaction cell death process induced by *P. triticina*. *TraesCS6A02G073300*, encoding a 3‐ketoacyl‐CoA synthase, has been reported to harbor quantitative trait nucleotides in close proximity to leaf rust resistance genes in wheat (Fatima et al., 2020). The 50S ribosomal protein L28 encoded by *TraesCS1D02G003700* belongs to the ribosomal protein family, and members of this family have been shown to confer tolerance against fungal pathogens in plants (Yang et al., 2013). Furthermore, *TraesCS7D02G217700*, encoding a glycosyltransferase, was highly and moderately expressed in the first leaf blade and leaf sheath, respectively, at the seedling stage. According to Bolton et al. (2008), two pathogen‐responsive genes encoding glycosyltransferases were shown to be upregulated under leaf rust infection. At the adult plant stage, *TraesCS1D02G004600*, encoding a cytochrome P450, was expressed in the flag leaf blade at both the dough and ripening stages. Different studies have reported the role played by cytochrome P450 in the host response to disease, which included the response to Fusarium head blight disease in wheat (Walter et al., 2008). The pathogen‐responsive gene encoding cytochrome P450 has been shown to be differentially expressed under leaf rust infection in wheat (Bolton et al., 2008). Additionally, Bolton et al. (2008) reported that gene models coding for the same protein as some of the hcmQTL discovered in this study were upregulated under leaf infection. All gene models coding for serine–threonine protein kinases and cytochrome P450 were upregulated in all treatments.

### 4.4 Breeding implications for leaf rust resistance

The primary use of MQTL for breeding purposes is the development of improved cultivars with enhanced yield that are resistant to diseases via MAS. Those MQTL with the smallest CIs have been harnessed effectively for MAS because they incorporate multiple QTL, as reported for disease resistance in maize (*Zea mays* L.) (Xiang et al., 2012; Wang et al., 2016), grain yield‐associated traits in rice (*Oryza sativa* L.) (Wu et al., 2016; Carrijo et al., 2017), seed quality in soybean [*Glycine max* (L.) Merr.] (Qi et al., 2017), and anthesis time in wheat (Griffiths et al., 2009). To this end, the MQTL were refined to 15 hcmQTL, each of them incorporating at least five original QTL and having a physical interval <20 Mb and a genetic interval <10 cM. In addition, MQTL analysis can be used to identify regions that confer resistance to more than one disease, and the marker information can be used for MAS (Ali et al., 2013). In this study, the hcmQTL1B.4 region was identified to confer resistance to leaf and stripe rusts, thus making it a potential region to exploit for multiple disease resistance in wheat. Furthermore, breeding for durable resistance is desired in major breeding programs. Durable resistance remains effective against a pathogen for a significant number of years (Johnson, 1981, 1984). The combination of seedling resistance and adult plant resistance has been proven to confer prolonged resistance over several years (Kolmer & Oelke, 2006). In addition, various studies have ascribed durable leaf rust resistance to adult plant resistance rather than to seedling resistance (Figlan et al., 2020). Therefore, hcmQTL1D.2, discovered in this study, can be harnessed to confer durable resistance in wheat, as it incorporates genes conferring both seedling and adult plant resistance. Another useful approach that could be harnessed in breeding for leaf rust resistance in wheat is gene pyramiding. Gene pyramiding involves incorporating multiple desired genes into a single cultivar. Gene pyramiding is broadly acknowledged by breeders, plant pathologists, and farmers to improve disease resistance in wheat (Chen & Kang, 2017). A major requirement for gene pyramiding is to identify various QTL or genes conferring resistance and then incorporate them into a high‐yielding cultivar (Singh, 1992). In several instances, this technique has been used in crops. For instance, long‐term resistance was conferred when diverse genes were pyramided with leaf rust genes (Kolmer, 1996; Bhawar et al., 2011; Aboukhaddour et al., 2020; Babu et al., 2020). Additionally, in barley (*Hordeum vulgare* L.), MAS combined with gene pyramiding has been used to introgress resistance loci against stripe rust into numerous lines (Toojinda et al., 1998, 2000; Castro et al., 2003a, 2003b; Richardson et al., 2006). To this end, the hcmQTL discovered in this study can be used and exploited for gene pyramiding via MAS to bolster the resistance of wheat against leaf rust.

### 4.5 Concluding remarks

One of the most effective methods for analyzing the wealth of QTL information available from various studies is MQTL analysis. In this study, we delineated the genetic architecture of leaf rust in wheat via MQTL analysis and by integrating genomics studies. Compared with initial QTL reports, meta‐analysis allowed us to reduce the MQTL CI, thereby facilitating the search for candidate resistance genes in the databases available. The result was the discovery of 15 hcmQTL, with each having a potential role in MAS. This result will be useful for developing resistance to leaf rust through the introgression of desirable hcmQTL that could confer a high level of resistance during cultivar development. Last, this study can also help better define the various mechanisms associated with leaf rust resistance in wheat.

## AUTHOR CONTRIBUTIONS

Aduragbemi Amo: Data curation, Formal analysis, Writing – original draft, Writing – review & editing. Jose Miguel Soriano: Conceptualization, Funding acquisition, Methodology, Project administration, Supervision, Writing – review & editing.

## CONFLICT OF INTEREST

The authors declare no conflict of interest.

## Supporting information

Supplemental Table S1: List of publications on past QTL mapping studies used for our meta‐QTL analysis.

Supplemental table S2: List of leaf rust resistance QTL identified in the mapping populations for meta‐QTL analysis.

Supplemental table S3: Leaf rust association studies used for MQTL validation.

Supplemental table S4: Validation of MQTL from previous GWAS on leaf rust in wheat.

Supplemental table S5: Co‐localization of leaf rust genes with MQTL.

Supplemental table S6: Discovery of candidate genes within the interval of hcmQTL.

Supplemental table S7: Differentially expressed genes from ERP013983.

Supplemental table S8: Differentially expressed genes from SRP041017.

Supplemental table S9: Differentially expressed genes from ERP009837.

Supplemental table S10: Gene ontology for genes expressed within the hcmQTL interval.

## Data Availability

All data supporting the findings of this study are available within the paper and within its supplemental materials published online.
